# Abstracts and Gleanings

**Published:** 1881-07-20

**Authors:** 


**Nature of Green Vomit.**—The chemical and microscopical nature of green vomit has been made the subject of recent investigations by Dr. F. Betz (Memorabilien, Oct. 6, 1880). It was supposed that the green vomit (*vomitus æruginosus*) was the result of a greenish discoloration of brown biliary pigment, caused by the acid action of the gastric juice. The supposition received corroboration from the bitter taste of the vomited matter, associated with its acid reaction. Dr. Betz concludes, however, that the green color is not invariably due to the presence of bile. The color often varies from a yellowish-green or grayish-green or dark-green, in accordance with the greater or less amount of the green "substances" and other admixtures. The green vomit may be kept for months and during the hot season, without spontaneous putrid fermentation taking place—a fact which militates against the possibility of its biliary or even animal nature. Dr. Betz also states that sometimes the green vomit has a natural or alkaline reaction. Microscopical examination shows the green substance to consist of an amorphous, finely granular greenish mass. Discoid heaps, or rounded colonies, are commonly observed. But the green substance may also form a lining over epithelial cells, salivary corpuscles, etc. From these facts Dr. Betz infers the vegetable nature of this substance, and he adds that it is probably derived from punctiform algæ, which he calls *chlorococcus.* He denies any relation of this low fungus to other microphytes, such as the torula cerevisiæ, sarcina ventriculi, or iodium. Finally, the author remarks that, apart from all other considerations, the frequent occurrence of copious green vomits would go to show that bile could not find its way into the stomach in such enormous quantity. The bitter taste of the green vomit receives its explanation in part from the frequent admixture of some bile, but is also in part due to the presence of a bitter principle in the chlorococcus.—*London Medical Record.*

### ABSTRACTS AND GLEANINGS.

**Remedies for Headache.**—The following recipes and suggestions for the treatment of different forms of headache are collected from a variety of trustworthy sources:

Two grains citrate of caffeine, in capsule, taken every half hour, is a very effectual remedy in nervous and sick headache. One or two doses are often sufficient to give complete relief.

The only objection to its use is sleepinesss, which sometimes results if it is taken in the evening. It is preferable to guarana, as being hardly ever rejected by the stomach.

The following, according to Dr. W. W. Carpenter, is very effectual in most forms of headache: Muriate of ammonia, 3 drachms; acetate of morphia, 1 grain; citrate of caffeine, 30 grains; aromatic spirits of ammonia, 1 drachm; elixir of guarana, 4 ounces; rose water, 4 ounces. Mix. Dessertspoonful every ten or twelve minutes.

In nervous headache, Dr. W. A. Hammond states the value of various drugs as follows:

Oxide of zinc is of great value. Odinary dose, 2 grains, three times a day, after meals; maximum dose, 5 grains. It is best given in form of pills.

Nux vomica is preferable to strychnia. The dose is ½ grain, after meals. If the patient be chlorotic, it is well to combine a grain of reduced iron and half a grain of sulphate of quinine.

Bismuth in the form of subcarbonate, will often take the place of oxide of zinc. Dose, two grains, after each meal. Bismuth probably aids digestion more than any mineral tonic, and is of use when there is gastric disturbance.

The bromides are serviceable when the nevous system has been irritated; when it is exhausted they do harm.

Phosphorus is very useful in most forms of nervous headache. The best results are obtained from dilute phosphoric acid, in doses of 30 drops, largely diluted, three times a day, after eating, or phosphide of zinc 1-10 grain, in pill, three times a day.

Arsenic as a nerve tonic stands next in value to zinc. Dose, 5 drops of Fowler's solution three times a day, after meals.

Galvanism is sometimes valuable, but by no means a specific. The *constant current* should always be used, being careful to avoid too great intensity, less amaurosis be produced.

Dr. T. Lauder Brunton, editor of the London *Practitioner*, says: The administration of a brisk purgative, or small dose of Epsom salts, three times a day, is a most effectual remedy for frontal headache when associated with constipation; but if the bowels be regular, the morbid process on which it depends seems to be checked, and the headache removed even more effectually, by nitro-muriatic acid, diluted 10 drops in a wine-glass of water, or bicarb. soda, 10 grains, in water, before meals. If the headache is immediately above the eyebrows, the acid is the best; but if it be a little higher up, just where the hair begins, the soda appears to be the most effectual. At the same time the headache is removed, the feeling of sleepiness and weariness, which frequently leads the patients to complain that they rise up more tired than they lie down, generally disappears.

A writer to the London *Lancet* remarks: At the Middlesex Hospital, female patients who have suffered many years from sick headache, evidently of a hereditary character, have been greatly benefitted, if not cured, by the administration of 10 minim doses of tincture of Indian hemp, three times daily before the attacks. This is well worthy of trial in those cases of ever-living, never-dying, martyrdom-like suffering.

In headache due to the determination of the blood to the head and in fever, the following simple treatment is to be commended: Put a handful of salt into a quart of water, add an ounce of spirits of hartshorn and half an ounce of spirits of camphor. Cork the bottle tightly to prevent the escape of the spirit. Soak a piece of soft cloth with the mixture and apply it to the head; wet the rag fresh as soon as it gets heated.

Soaking the feet in very warm water, in which a spoonful of mustard has been stirred, is also beneficial in drawing the blood from the head. Two teaspoonfuls of powdered charcoal, well stirred in half a glass of water, and drunk at once, is a valuable remedy in sick headache from sour stomach, flatulence, etc.

Tincture of nux vomica is recommended by Ringer as possessed of real curative powers, when given in drop doses, repeated every 5 or 10 minutes, for 8 or 10 doses, and then continue at longer intervals, for sick headache, accompanied with acute gastric catarrh, whether due to error in diet, constipation, or no apparent cause.—*Boston Journal of Chemistry.*

**Cerebro-Spinal Meningitis.**—Dr. Cleveland (Academy of Medicine, Cincinnati,) reported a number of cases in his practice of such a nature as to suggest to him that perhaps cerebro-spinal meningitis would be a timely subject to introduce before the Academy. But before reporting the cases which he had, he would wish to make a few introductory remarks on the general subject of cerebro-spinal meningitis. This disease, while we must believe that it has existed from time immemorial, is new in history. Our knowledge of it dates back only to the middle of the last century, and it is only since the present century that this disease has been thoroughly studied and worked up. But the result of these investigations have been unsatisfactory so far as giving us a thorough understanding of this disease. As to its etiology, a host of predisposing causes have been given and surmised; but no one of them is constant and holds good in every case. It attacks all classes; those with the best hygienic surrounding as well as the worst. It attacks indiscriminately city and country, hot and cold climates, and at all seasons of the year, though our epidemics have generally been in the spring and fall.

The inception is generally sudden, with very few or no premonitory symptoms. Headache, supra orbital, occipital, or basilar, are almost constant symptoms; pain in neck, and stiffness of the muscles of the neck; pain in the back, and reflected to the extremities, of a very severe character; delirium is not a constant factor, though it sometimes occurs; nausea and vomiting very frequent; bowels usually constipated.

In some epidemics a petechial rash is always found, though eruptions of different kinds may appear; in other epidemics no eruption is found. Hyperæsthesia is also a marked feature of the disease. The mortality in this affection is always high, and in some epidemics very high. The average mortality is about 25 per cent., though it has run as high as 75 per cent. The prognosis then is always grave.

In speaking of this disease it has been remarked by some one that it is "easier to say what it is not than what it is." Some regard it as simple acute cerebro spinal meningitis. This view is obviously untenable. The point of attack is the cerebro-spinal meninges, but the violence of the disease is often out of proportion to the pathological changes of these parts. The view has been long held that it is but a variety of typhus, and there is much in the symptoms of many cases to encourage this view, but of course this theory will not hold good now. It has been, and is still, regarded by some as pernicious malarial fever. It has been explained by some by calling in the aid of epidemic influence: a mild epidemic producing influenza; a more severe one typhoid-pneumonia, a malignant influence producing cerebro-spinal meningitis.

That it has a specific cause is generally believed, but its exact nature, whether it be a germ or a poison, has not been ascertained.

The first case that attracted his attention was C. Z., æt. 20, attacked March 24. Severe pain in back of head, neck, and spine, extending into the extremities; muscles of neck becoming stiff on the second day after the inauguration of the attack. Nausea and vomiting, or rather vomiting with a very little nausea; pulse never run above 80 when observed; temperature never above 101°.

*Treatment*—Morph. hypodermically, and quinia and potassii iodidi per os. Was kept constantly under morphia for eight days. When not sufficiently under the drug, pain in the head and neck intolerable. Recovered after two weeks. His neck is yet a little stiff, and he is yet troubled with some headache.

Case 2. T. F., April 4th, suddenly taken, while in apparent good health, with vomiting and convulsions. The next day his head was thrown back, muscles of neck rigid; cried with pain if moved, tenderness along the neck and spine; pupils contracted, eyes closed, brow corrugated, hands tremulous. The child had three separate convulsions at intervals of about twelve hours.

*Treatment*—Opium in sufficient quantity to keep down pain, with quinia and potassii iodidi. After twelve days convalescence appeared to have set in, and at present the child seems to have recovered.

On the eighth day of this case a rubeolous eruption appeared in spots all over the body, which in twenty-four hours disappeared.

Case 3. April 4th, taken in apparent good health with convulsions (temp. 103°, pulse 110) and vomiting, which never let up; died in twenty-four hours. This may not have been cerebro-spinal meningitis, but the inception of the disease was so exactly similar to case No. 2, that the speaker had given it for what it was worth.

Case 4. J. W., æt. 10. Taken April 7th with chill and vomiting. This recurred the next day. Pain in the head, neck and back; wildly delirious from the first; screams "my head! my back!" Lies on his side, with head thrown back; brow contracted as if in pain; pupils small, eyes closed tightly, hands tremulous, and vainly grasping at something; screams with pain if moved, and also whenever the effects of the opiate wears away.

*Treatment*—Quinia and bromide of potash, with opium as pain and restlessness requires. This case is still under treatment, with the prognosis very gloomy. On the 8th or 9th day some hyperæmic blotches appeared on the face and neck. No petechiæ were observed in any of the cases.

Cases 5 and 6. One was taken April 8th, and the other April 10th. They both occurred in the same tenement house. The course and symptoms were so similar that they could be reported together. Both commenced with recurrent chills and fever, vomiting pain in the head, neck and back; and remittent fever with opisthotonos and stiffening of the neck. The pain was not sufficient to require opium in these cases. They were kept on potas. bromide and quinia from the beginning. Both are convalescing and apparently will recover. Case 5th, at the end of the first week, had blotches of a herpetic eruption on the face and neck. It is true that cases 5 and 6 were very mild in their inception and course for cerebro-spinal meningitis; but it must be borne in mind that this disease occurs in all grades of severity. Cerebro-spinal meningitis certainly exists in our midst. The appearance indicates that we are probably on the eve of an epidemic. If the accumulated filth of a long winter has anything to do with the development of this disease—and it surely has—we have in our midst all the factors (heat, moisture, animal and vegetable filth) for breeding this and kindred maladies.—*Lancet and Clinic.*

**Retained Placenta.**—Dr. H. Frost, of Salem, Va., in *Medical Bi-Weekly*, says: I was summoned, several hours after the birth of the child, to deliver the placenta, which I found closely adherent. The cord had been torn from its attachment. Finding it impossible to remove the whole, I took away as much as I could, about half. I gave her at once quinine, grs. v., every three hours, and used vaginal injections of carbolic acid two or three times a day, and intra-uterine injections of the same at my daily visits. Gave also salicylate of soda. Each day I endeavored to remove the mass, but failed. On the fourth day the placenta was expelled, and the patient recovered without further illness.

I have met with four other cases in which the placenta was firmly adherent; three of them, strange to say, in the same family, and two in the same patient. In each case, after my own efforts failed, I had the advice and assistance of other physicians. In one, after long continued efforts, the placenta was removed, but the woman died of septicæmia. In each of the other three the placenta was expelled spontaneously on the third or fourth day; one patient died, the others recovered with no illness whatever.

These cases, it seems to me, show that where the placenta is so closely adherent that it cannot be delivered without the desperate efforts which are often made, it is better to leave it for nature to expel, guarding the patient by appropriate remedies against the dangers of septicæmia.

I have been led to report the above cases from hearing an old physician say recently that no patient of his should die with a retained placenta; he would prefer that she should lose her life in his efforts to deliver it. It is no doubt a most distressing alternative we are called on to take, and we feel that if we leave a patient with her labor thus incomplete we may subject ourselves to reproach, but my experience, limited though it be, convinces me that life is often destroyed by the ill-advised force which is used. It is common to hear doctors say that they never saw a placenta so adherent that they could not remove it. They are more fortunate or more skillful than I, for in each of the cases mentioned above in which the placenta was left in the womb, I found it impossible to feel the placental margin—to determin where it ended, and the uterine surface began. In such a case what is to be gained by tearing it away piecemeal? Are we not sure to leave some portions still adhering? And would not the retention of a small portion be as dangerous as the retention of the whole mass? Would it not be *more* dangerous? For the whole placenta by its presence provokes the womb to constant expulsive efforts, while the small portion, being better tolerated, would probably be retained longer and thus expose the patient for a longer time to the dangers of blood-poisoning.

I hope I will not be understood as advocating inaction in this serious complication of labor. By no means. Let us use every reasonable effort to remove the placenta, but let us be careful not to push those efforts too far. Many lives have no doubt been sacrificed from a conviction on the part of the attendant that the placenta must be extracted *at all hazards*. May the report of these cases induce some to let their patients alone before they inflict injury on them.

**An Important Modification of the Ordinary Anæsthetic Method.**—The Revue de Chirurgie et Arch. Med. Belge observes that Messrs. O. de Stefanis and Vachetta, convinced of the dangers attending the use of anæsthetics—statistics showing that with chloroform there occurred one death in 2873 cases; with ether, one in 23,-204, while bichloride of ethylene showed one death in 5006 cases—propose a modification, which has already given them excellent results, both upon men and animals.

It was suggested to them by this consideration, that death being caused by cerebral anæmia or cardiac paralysis, anything that could induce a congestion of the nerve centres and excite the heart would help in controlling the noxious effects of the anæsthetic agent, without diminishing insensibility. For this purpose they make use of alcoholics. We shall not repeat here their experiments upon dogs and rabbits; it is more interesting to note the method recommended to bring on inebriety in the subjects to be anæsthetized.

In place of prescribing a strict fast, they advise patients to eat a light breakfast of crackers or bread, and according to age, sex, strength, and the habits of each one, to drink a certain quantity of some light wine, like claret. This quantity may vary from 100 to 200 grains, (f. ʒ xxv–f. ʒ l.) Those accustomed to alcohol may besides take a little brandy. When the heart is found to be sufficiently excited, the inhalation of ether or chloroform may be proceeded with.

Experience proves that under those conditions complete anæsthesia is obtained in a few minutes, by the use of from five to ten grams

(*m* 75 to 150), of the anæsthetic agent. Judging from their first experiments, the authors believe themselves warranted in drawing the following conclusions:

When a dog is prepared for anæsthesia by the aid of Marsala wine, he is less sensitive to the effects of ether or chloroform, but the dangers inherent to anæsthesia are reduced. If the animal is inebriated, insensibility and muscular relaxation are longer in showing themselves.

If anæsthesia is repeated at short intervals, the subjects become less liable to its effects. Alcoholic intoxication, both in man and in animals, does away with all danger arising from cardiac paralysis, or cyanosis due to vaso-motor palsy; no emesis has ever been noticed.

A man slightly under the effects of alcohol yields more readily to an anæsthetic; a lesser quantity is required to induce sleep.

In no case has sleep been followed by such marked secondary phenomena, like vomiting, prolonged somnolency, and falling temperature, as happen with patients who have been put to sleep by the ordinary method.—*Med. and Surg. Reporter.*

**Sunstroke and its Treatment.**—Dr. D. H. Cullimore writes to the British Medical Journal:

"I landed in India an orthodox believer in the absolute necessity of rapidly attempting to reduce the body temperature by cold baths, and in two forms of sunstroke I am still of opinion, mainly on theoretic grounds, that the treatment is the most effectual we possess. There is, however, a third form, and one that most frequently comes under the notice of the medical officer in India—at all events while in civil employment—in which my experience has not only taught me to prefer the tepid and warm baths (from 90° to 98° Fahr.), but has led me to think that cold baths proved rather injurious than otherwise. This third form was probably the disease, complicated with malarial fever, from which the Marquis of Ripon has lately recovered. The varieties of sunstroke to which, in my opinion, the cold bath should be restricted are these:

"The first is the sudden stroke from the direct effect of intense sun heat, combined with great fatigue, and predisposed to, perhaps, by the use of stimulants. This form is rapidly fatal; it most frequently occurs in young, vigorous, unacclimatized men, whose internal organs are probably sound; and is attended with loss of consciousness, pungent heat of skin, perhaps convulsions, and death from syncope, owing either to stunning of the brain or to paralysis of the conducting nerves and their centres, brought about by a coagulation of the albuminous bodies in the nerves, muscles, etc. Here the immediate and repeated use of the cold bath, with the application of cold to the head, seems rational enough. I have seen but one case of this kind; and death was of too rapid occurrence to allow any treatment to be adopted with any chance of success. There were *post-mortem* signs of cerebral congestion and effusion of blood. The lungs were considerably engorged, The patient had an epileptic history.

The second form, in which the immersion of the whole body in cold water will reduce the temperature so as to permit the renewal of the suspended functional activity of vital organs, is that kind of heat asphyxia known to occur on board of ship in narrow tropical seas, or ashore in the crowded barrack-room.

The third variety, and that in which my experience has led me to discard the cold and adopt the tepid and warm bath, may be described as follows: It occurs most frequently among acclimatized district civilians—engineers, police and medical officers—men whose duties necessarily expose them, at times, to great and prolonged heat, considerable fatigue, and a good deal of discomfort, while sojourning in tents or travelers' bungalows. They are probably tainted with malaria, and may have occasionally suffered from attacks of congestion of the liver and dysentery. While on a tour of this kind, the patient elect begins to feel irritable, tired, and out of sorts; he tries to look bright and pull himself together. After a day or two, the heat of skin increases, and he ceases to perspire; there are headache and intolerance of light; and when considerably done up he returns home, and after a sleepless night sends for the doctor. His face is now flushed; there is intolerance of light and sound; perhaps delirium and muscular twitches; the skin is dry and burning; the temperature 106° or 107° Fahr, with exacerbations if complicated with fever. The pupils are often contracted, and there may be tenderness over the hepatic region, with a yellow conjunctiva. Patients suffering as described may recover if treated promptly. The disease is liable to recur, and a sojourn in Europe is advisable, but not absolutely necessary.

The treatment which I have adopted in several cases of this affection, and to which, were I a patient myself, I should wish to be subjected, is as follows: A warm bath, to be repeated according to the judgment of the medical attendant; cold to the head, in the form of irrigation, if the patient will bear it; and removal to a eool, dark room, with a punkah, A thermantidote would be a great advantage; it is, however, necessarily restricted to public institutions, and I have never seen one in use in India. Aconite and belladonna, in from three to six minim doses, should be given every two hours. This combination is invariably followed by free perspiration, but a coincident reduction of the temperature does not always accompauy it. Still it is the best means of attaining that end, at the same time controlling the meningeal disease. Bromide of potassium is a useful addition in some cases; chloride of ammonium in others; and quinia, if there be a malarial complication. Quinia. unless in cases of ague, does not, I think, reduce the temperature of the body. Potash water is the best beverage.—*Med. and Surg. Reporter.*

**Interval Between Apparent and Real Death in Asphyxia from Lack of Air.**—M. Laborde has been investigating a question of great importance as well from a physiological as practical standpoint; one that, notwithstanding the numerous researches in regard to it since the memorable labors of Beihal up to those of our president, M. Paul Best, still remains unsolved. .

It is the interval between the apparent and real death of animals asphyxiated by deprivation of air.

It is this type of asphyxia chosen by M. Laborde in order to always have as nearly as possible the same conditions. The animal being tracheotomised, a Bichah canula is introduced into the trachea, and the cock closed. The time for the supervention of the apparent death varies largely according to circumstances; a medium, however, can be established, amounting to from eight to nine minutes.

At this point there occurs first the cessation in the respiratory movements of the chest, then the dilatation of the pupil, and finally insensibility of the cornea. Ordinarily the cardiac movements persist—diminishing more or less in frequency and strength.

The cock may be opened, the blood does not flow less dark.

If artificial respiration is resorted to, there will be seen at the end of two minutes the acceleration or resumption of the cardiac movements, at the same time contraction of the pupil; the latter, however, not a constant occurrence; then the sensibility of the cornea returns and the animal breathes spontaneously.

If, after a time, the experiment is repeated upon the same animal, the phenomena attendant upon restoration occur in the same order, but after a much longer time. It is necessary to keep up the artificial respiration for twelve minutes instead of two.

After the dog breathes spontaneously through the open canula, the pupil that was contracted from the first artificial respirations, assumes its normal state.

Artificial respiration is not the only means of restoring life to the animal, since excitation of the pneumogastric will produce the same result.—*Lancet and Clinic.*

**Physiological and Therapeutic Action of Benzoic Acid.**—The *Lancet* states, that from his experiments Schulte has found that the introduction of benzoic acid into the stomach of warm and cold-blooded animals always causes irritation of the mucous membrane, extravasation, and hemorrhagic erosion, and that similar irritation is sometimes observed even when benzoic acid or its salts have been introduced by injection under the skin, or into a vein. In the latter case the pulse and respiration are at first accelerated, and afterwards retarded. The blood-pressure is not influenced by slight injections, but is lowered by stronger ones, and the phenomena are independent of the vagus nerve. Whenever the dose amounts to more than two-thousandths of the body weight of the animal, toxic symptoms occur, followed by death. Salkowski has pointed out that when benzoic acid is given, the urine commonly contains a substance which is capable of reducing sugar. This body is not soluble in ether, but is soluble in alcoholized ether and acetic acid. It is very slightly soluble in water, and thus is distinguished from both benzoic acid and hippuric acid. The salt it forms with barium is insoluble in water, and contains both nitrogen and chlorine. Schulte has found that the appearance of this substance in the urine corresponds in time with the appearance of toxic symptoms. In the therepeutic use of the benzoic acid and benzoate of soda for diphtheria, acute and subacute articular rheumatism, erysipelas, typhus, diabetes, nephritis, interstitial phthisis, peritonitis, etc., the reducing substance was never found in the urine after a small subcutaneous injection, but was found when a large injection was given. The salt was found distinctly useful only in acute articular rheumatism and diphtheria.—*Michigan Medical News.*

**The National Board of Health and the International Sanitary Conference of 1881**, was the title of a paper read by Dr. James L. Cabell, of the University of Virginia, and President of the National Board of Health. He alluded to the illiberal action of the National Congress in reference to the National Board of Health, and spoke of the success that had attended the efforts of New York physicians for the establishment of a State Board of Health. He also gave a history of the establishment of the National Board, and the reasons why it had not secured sanitary regulations in Stat s which had not established State Boards; and stated the reasons which impelled the calling of an International Sanitary Conference. He gave a very interesting account of the proceedings of that Conference, and the good work accomplished by it.

After dwelling for some time upon the work prepared at the Conference, Dr. Cabell concluded as follows:

"There is, therefore, good reasons for hoping that an international agreement may be arrived at between the States more frequently threatened with epidemic invasions. And, aside from this, the degree of attention which, as a result of the deliberations of the Conference, has been given to the subject of maritime sanitary police, cannot be without fruit in securing greater cleanliness, better ventilation of ships sailing on the high seas, and in general an improved sanitary condition of these important instruments of commerce, which become so often the carriers of the most deadly contagion, from the failure to use such precautions as sanitary science suggests, and as it is hoped will now be enforced among the maritime powers of the world."—*Va. Med. Monthly.*

**Earache.**—In the American Medical Association, Dr. Jacobi remarked that closing the mouths of infants and children, and simply blowing into the nose, is often a very valuable method of relieving severe earache, and that in a number of cases he had obtained most excellent results from this procedure, the cause of the troub'e probably being a catarrhal affection of the Eustachian tube.

Dr. LEIGH, of Petersburg, reported two fatal cases of poisoning from chlorate of potash. The first was of a child three years of age with diphtheria. The mother gave the child one half ounce of chlorate of potash, and the child died within 24 hours.

The second case was a child five years of age with croup. It took six drachms of chlorate of potash in 24 hours; cyanosis appeared in six hours; hæmaturia and death in 48 hours.

Dr. FONTAINE knew a patient who took one ounce and died. Chlorate potash is soluble in 18 or 20 parts of water. Dr. J. does not give more than a grain to a child under one year, or 1½ to an adult in 24 hours.

**Cancer of the Tongue.**—In the December number of the Bull. et Mem. de la Soc. de Chir., is a report of a communication by M. Verneuil, on the inutility and danger of any salve operative treatment of epithelioma of the tongue. This disease, it is pointed out, has never been cured either by internal treatment or by topical applications.—*Med. News and Abstract.*

**Gastric Remittent Fever.**—In confirmation of the views respecting this disease advanced by Dr. F. Peyre Porcher, in the January number of the American Journal of the Medical Sciences, we publish the following from Dr. D. I. Cam, of Ashville, N. C. :

"The disease is essentially '*gastric.*' The bowels, if at all, are only slightly involved. It is an irritation, not an inflammation. This is the true pathological state. My study of this disease, which has been deep and extensive, does not lead me to agreement with the view expressed, or at least suggested, by Dr. Charles West, that it is the child's typhoid fever. There is one sign of the disease which I consider as nearly *pathognomonic*, so nearly invariable is it. This is the child lying with the eyes obstinately closed, only opening them when aroused by being shaken or loudly spoken to.

"As regards the treatment, I yet pursue, substantially, the course which you mention; but I now use bismuth and oxalate of cerium every three or four hours, considering these are among the best means we have of allaying gastro-intestinal irritation; and I use injections of warm water and assafœtida p. r. n. to move the bowels—not insisting upon purgation as an important part of the treatment. The mortality must be infinitesimally small. If I have ever lost a case, I cannot remember it.

"The disease is as common here as it is on the seaboard or elsewhere."—*Medical News and Abstract.*

**Thapsia Plaster.**—This plaster is made from the *Thapsia garganica*, which grows in abundance on the plateaus of Algeria, and since its introduction into therapeutics has rendered important service in the art of healing. It owes its irritating properties to a peculiar resin contained in its roots, rendering it a powerful derivative, whose action, on account of its form and consistence, may be graduated at will and limited to the very spot on which the physician wishes to obtain the effect. This result is impossible with croton oil, which on account of its fluid state spreads and acts on a larger space than desirable. From the simplicity of its use it has gained much favor with the profession, and is used by many in preference to croton oil or tartar emetic ointment, over which it has great advantages. Its revulsive action manifests itself by a miliary eruption more or less abundant according to the time it has been applied. When the eruption disappears in a few days, the plaster may be reapplied, and thus a counter-irritation kept up for any desirable length of time.—*Pacific Medical Journal.*

**Lemon Juice in Diphtheria.**—Dr. J. R. Page, of Baltimore, in the New York Medical Record, May 7th, 1881, invites the attention of the profession to the topical use of fresh lemon juice as a most efficient means for the removal of membrane from the throat, tonsils, etc., in diphtheria. In his hands (and he has heard several of his professional brethren say the same) it has proved by far the best agent he has yet tried for the purpose. He applies the juice of the lemon, by means of a camel's hair probang, to the affected parts, every two or three hours, and in eighteen cases in which heh as used it the effect has been all he could wish.—*Med. and Surg. Reporter.*

**Benzoate of Sodium in Acute Rheumatism.**—Dr. Davis-MacEwen (*Brit. Med. Jour.*) observing that benzoic acid is closely similar to salicylic acid in chemical composition, and somewhat the same in physiological effects, endeavored to determine whether it, like the latter, possesses anti-rheumatic properties. He publishes notes of five cases in which the remedy was employed in the form of benzoate of sodium. On the first occasion in which he used it, the relief of pain and subsidence of fever were so immediate, and the recovery was so rapid and complete, that he had no hesitation in adopting the same treatment in subsequent cases. The dose was, in the earlier cases, fifteen grains of the salt every three hours; in the later cases, twenty grains every two hours. In all the cases the symptoms passed off in periods varying from three days to a week after the commencement of the medication; in none did cardiac complications arise in the course of treatment, and Dr. MacEwen thinks the convalescence was more rapid than in cases he had seen treated with salicylate of sodium. Benzoate of sodium possesses this advantage, that it does not give rise to the nausea and depression or the unpleasant head-phenomena which the salicylate frequently produces. It is most conveniently prescribed in the form of a mixture, and it may be given in doses of fifteen to twenty grains every two or three hours. It should also be continued in diminished doses for twenty-four to forty-eight hours after the rheumatic symptoms have disappeared.—*Med. Times.*

**How to Vaccinate.**—Dr. Dozier (in *Medical Independent*) says: I use *only fresh bovine virus;* and prefer the ivory points to any other. I dip the point to be used in *cold water*, then lay it aside, in order that the virus coating may become well dissolved or softened. In the interim I scarify with a dry ivory point, stroking very lightly, scraping off as completely as possible, *without bringing blood*, the cuticle or scarf skin, for a space of about a quarter of an inch square. I then rub over the abrasion the previously prepared point, until the virus is all off, and insist on the sleeve being kept up for at least five minutes after the operation. I then place over the part a piece of adhesive plaster of sufficient size to cover well the scarification. One of two hundred and twenty-five vaccinated adults and children, and many of the former having been successfully vaccinated before, I got a result of *ninety-seven* per cent. of successful vaccinations! While my colleagues, who used lancets, principally, with which to scarify and *cut* rather than *scrape* through the outer skin, consequently causing more or less bleeding, were successful in not more than fifty per cent. of their cases.

**An Old Treatment of Dysentery Revived.**—Mr. Henry Colley, in *Med. Times and Gazette*, says that in the ordinary dysentery of adults with some fever, much griping, constant attempts at stool, with tenesmus, the evacuations consisting of bloody and shreddy mucus, without any proper fecal matter, the proper procedure is to give every half-hour half a minum of the liquor hydrargyri bichloridi (B. P.). The first dose will sometimes relieve the pain; in a few hours the tenesmus ceases, and on the second or third day healthy stools make their appearance. Mr. March looks upon the remedy as the perfection of medication as a specific tonic.

**Chloroform in Diphtheria.**—Dr. Lathrop, of New Hampshire, at American Medical Association, said that he had experimented with chloroform largely, and finds it a highly useful agent. He uses it in diphtheria and other throat affections, applied on a piece of cotton attached to a tube or pen-holder. The patients usually required visiting no longer than four days; but the cases were not so malignant as had been reported in other localities.

No unpleasant effects have ever followed this plan of treatment, and the child, in true diphtheria, does not complain of *smarting* from the application of chloroform. He had used this plan of treatment in one hundred cases. Of course constitutional measures are added.

Dr. McNeal, of Gettysburg, Pa., recommends the following: Potass. bromid., ʒj; potass. chlorat., ʒij; acid. carbolic., gr. xx; aquæ, Oj. Use in an inhaler. Locally, chloroformi, ʒij; lin. saponis, ℥j–ʒij.

Dr. F. E. Hitchcock, of Rockland, Maine, uses equal parts of sulphurous acid and water in an atomizer. The proportions can be varied, and the acid used as a gargle, with cold effusion externally.—*Atlanta Medical Journal.*

**Propagation of Syphilis by Razor.**—M. Despres, in *Journal des Connaissances Medicales*, has lately published two cases in which syphilis appeared to have been communicated through the medium of the razor during the process of shaving. In the first case, a man aged fifty-four, of steady habits, and with no history of venereal disease, was shaved by a barber on July 11, 1880. The man observed, after being shaved, that he had three small cuts on the chin. On July 25, the patient, who had had no relation with women for ten weeks, noticed a swelling at the site of each of the cuts first noticed after the shaving. On September 1 the patient came under the care of M. Despres, having been sent to that surgeon as a case of epithelioma. On examination there were found three ulcers on the chin, surrounded by some red and moderately hard callosities. There was a hard gland beneath the jaw, but none elsewhere. No other signs of syphilis were discovered at that time. On September 15 a papular syphiloderm appeared.

The second case, that of a young man aged 22, was in many respects similar to the preceding. In him also the initial lesion appeared on the chin, but the patient did not remember having been cut by the razor. In due time glandular enlargement and a general syphiloderm appeared.—*Med. Times.*

**The Death Smell.**—Dr. A. B. Isham, professor of Materia Medica and Therapeutics in the Cincinnati College of Medicine and Surgery, calls attention in the *American Journal of the Medical Sciences* for April, 1881, to the peculiar ante-mortem odor encountered in many cases at a variable period before the fatal result; in one case he noticed it thirty-three hours before death. The smell is analogous to musk, but is rather more pungent and less diffusible. He is inclined to attribute the phenomenon to the liberation of ammonia, and of the peculiar volatile oil (fatty acid) which gives the blood its odor; this liberation being caused by the diminishing vitality of the blood.—*Michigan Medical News.*

